# Addressing neonatal intensive care unit clinicians’ distress via interprofessional meetings

**DOI:** 10.1038/s41372-026-02763-1

**Published:** 2026-06-22

**Authors:** Katharine Press Callahan, Katie Behringer, Douglas Hill, Kimberly Mason, Jennifer Walter, Lara Lechtenberg, David Munson, Chris Feudtner

**Affiliations:** 1 The Children’s Hospital of Philadelphia, Philadelphia, PA USA; 2 Department of Medical Ethics and Health Policy, The Perelman School of Medicine at the University of Pennsylvania, Philadelphia, PA USA

**Keywords:** Medical ethics, Paediatrics

## Abstract

**Objective:**

Our primary objective was to determine whether brief, as-needed interprofessional meetings reduced distress related to specific patient cases for interprofessional clinicians in the neonatal intensive care unit.

**Study design:**

We collected pre- and post-meeting surveys to quantify clinicians’ distress.

**Result:**

We collected 416 paired responses (80% participation rate) from 192 unique clinicians.

**Results:**

After meetings, clinicians reported reduced intensity of most distressing thoughts; they were more likely to feel their perspective was heard (1.51 to 1.17, SD 0.82, *p* < 0.001), the team was united (1.56 to 1.22, SD 0.84, *p* < 0.001), they could provide good care (1.76 to 1.62, SD 0.98, *p* < 0.001), and that the rationale for care was reasonable (1.93 to 1.52, SD 0.93, *p* < 0.001). Moral distress was also lower on post assessments (5.47 to 5.27, SD 2.55, *p* = 0.005).

**Conclusions:**

Brief, as-needed interprofessional meetings decreased distressing thoughts about challenging cases, particularly for those experiencing the greatest distress.

## Introduction

Work-related distress takes a toll on clinicians in the neonatal intensive care units (NICUs) [1, 2]. Causes of distress range from internal beliefs of clinicians to larger systemic and policy issues. Moral distress results from a clinician being constrained from doing what they believe is morally correct [3, 4]. Concerns without an overt moral dimension also contribute to distress [5–7]. Clinicians may be upset by challenging care tasks or when their perspective is not acknowledged by teammates [8]. Large, rotating clinical teams pose communication challenges [7, 9]. The omnipresence of death in the NICU causes understandable sadness among clinicians [2, 10]. Clinicians’ suffering is a problematic end in itself and can also lead to poor job retention [11] and adverse patient outcomes [11–13]. At times, clinicians’ distress becomes so unmanageable as to impede day-to-day patient care [7].

Given the sadness inherent in everyday NICU care, alleviating all distress is likely not an achievable, or perhaps even appropriate, goal [14, 15]. Yet, interventions to help keep distress from reaching overwhelming levels are needed to support clinicians in taking care of themselves and their patients. Researchers have developed many approaches for distress, paralleling the varied causes of distress. Helping team members with less power advocate for their opinions [16, 17], increasing knowledge about ethics and decision-making frameworks [18, 19], and facilitating self-reflection [20] can help alleviate moral distress. Organization-wide reflective case discussions decrease distress over time [21, 22]. More recently, researchers have theorized and trialed targeted, responsive programs to support clinicians through particularly distressing cases [7, 21]. Such programs could support clinicians when they are most vulnerable and minimize the impact of clinician distress when patients are most at risk. Reflective programs have a theoretical basis in cognitive behavioral therapy, one of the most effective approaches to mitigating distress [23, 24]. Both strategies promise to interrupt a cycle of distressing thoughts and behaviors by providing a structured opportunity to reexamine thoughts and replace them with more positive ideas. Yet, to our knowledge, no such programs are validated to help intensive care clinicians.

This paper reports the development and evaluation of a program that facilitated as-needed case-specific meetings to support clinicians—medical, nursing, and beyond—in the neonatal intensive care unit (NICU). Similar to other reflective practice interventions, the objective of the meetings was to use structured reflection to elucidate the reasons for distress, reflect on the case in an open-ended way, and consider existing solutions [21]. We used pre-and post- evaluations to assess the impact of meetings. Both the program design and evaluation were based on a conceptual model about the causes and consequences of clinicians' distress, with the model being refined over the course of running the program.

## Methods

### Program development

The “Pink Flags” program started in the fall of 2020, and the program name was chosen to reflect our hope that earlier intervention could prevent a pink flag from turning red, diffusing tensions before distress became unmanageable. Since its inception, a nurse educator (K.B.), a nurse ethicist (K.M.), and a neonatologist with ethics training (K.P.C.) have co-led the program. This study represents an evaluation of the program several years after its establishment.

Through the Pink Flags program, any member of the care team could request an interprofessional meeting to discuss a distressing case. Requestors emailed program leaders, who arranged meetings to ensure that the attending neonatologist, charge nurse, and social worker could attend. We organized meetings in real-time, within 5 days. We invited the entire team caring for the patient, including social workers, physicians, nurses, nurse practitioners/physician assistants, chaplains, music and art therapists, and respiratory, speech, occupational, and physical therapists. We invited subspecialty teams who were intimately involved with cases. Meetings were 45-min and occurred virtually. One of the three members of the Pink Flags leadership team served as the facilitator, and the additional leaders attended when possible.

The primary intervention of the Pink Flags program was to coordinate and support a space for team members to speak openly. The purpose of meetings was not to alter medical plans or provide specific education but instead to facilitate communication and mutual support. The facilitator initially asked the attending physician, social worker, and bedside nurse to share perspectives on the case (meeting guide provided as Supplementary Fig. 1). Then, we dedicated most of the time to discussion and structured reflection [25, 26] in which the team considered the causes of distress and existing ways to manage them. The facilitator typically spoke minimally, interposing reflective or supportive statements. The facilitator concluded meetings by reviewing and summarizing with an emphasis on any strategies the group suggested for managing distress. At least one Pink Flags leader kept detailed field notes at each meeting. These notes were used to send a meeting summary to care team leaders and were reviewed for research purposes.

### Study design and participants

We collected pre- and post-meeting surveys from individual participants. We powered the study to detect 10% decrease in distress. We used data from the original validation of the moral distress thermometer instrument [27] to guide sample size calculations, calculating a minimum sample size of 215. We conservatively set our target at 250–300 paired responses and planned to collect data at 25 meetings.

All clinicians who attended Pink Flags meetings in their entirety were eligible to complete questionnaires. Questionnaires were distributed using REDCap [28] links and QR codes at the beginning and the conclusion of each meeting. An embedded statement of consent preceded participation. At each meeting, at least one member of the study team took field notes, recording key aspects of the discussion.

### Ethics approval and consent to participate

Our study was approved by the institutional review board at the Children’s Hospital of Philadelphia. All methods were performed in accordance with regulations; all participants provided consent.

### Conceptual model development and refinement

Over the course of this project, the study team developed and iteratively revised a conceptual model depicting the origins and consequences of clinicians’ distress. We constructed the original model by reviewing literature on the psychology of distress [23, 29], moral distress [3], and cognitive behavioral theory [24, 30]. We then revised the model based on the study team’s review of notes taken at 66 Pink Flags meetings preceding initiation of this research. We coded meeting notes to extract the five most common distressing thoughts that clinicians expressed, here followed by abbreviations used throughout this manuscript: The case makes me feel sad (“sad”); I feel my voice is not heard by the care team (“voice not heard”); I do not feel good about the care I am providing (“not good care”); The rationale for this patient’s plan is not reasonable (“rationale not reasonable”); Our team does not feel united in taking care of this patient (“team not united”). The model served as the basis for the design of the pre- and post-meeting questionnaires (Supplementary Figs. 2 and 3).

We used qualitative analysis of the field notes to revise the conceptual model components and re-assess relationships between the components in a rolling, ongoing manner.

### Quantitative measures

#### Distressing thoughts

We evaluated the impact of the interprofessional meetings on the most common distressing thoughts cited above. We converted each of the five thoughts into a Likert-scaled item that participants could assess from “strongly disagree” to “strongly agree. We used or adapted previously validated questions (See Table 2 for items, citations are provided in Supplementary material).

#### Moral distress

We included the moral distress thermometer, a validated scale of moral distress [27], and a standard definition, “feeling you know the right thing to do but are unable to do it” [3, 4].

#### General negative reactions

In a separate question, we adapted the moral distress thermometer to measure “any negative reactions to the patient case.”

#### Meaningfulness of work

We included a validated measure of finding one’s work meaningful, elsewhere found to reflect resiliency to work-related distress, in the pre-meeting questionnaire only. We planned to evaluate this as an effect modifier to test the hypothesis that people who found their work more meaningful may reap more benefit from meetings [31].

#### Demographic measures

In the post-meeting questionnaire, we collected information about clinicians’ professions and years in practice. We used self-generated identification codes to link pre- and post- meeting questionnaires [32].

We piloted the questionnaire with six clinicians from varied professions to ensure questions were comprehensible and understood as we intended.

### Quantitative data analysis

Quantitative analysis was performed using Stata 19.0 (College Station, TX). We ensured the data were normally distributed, then assessed differences between pre- and post- meeting responses using paired t-tests. We calculated effect sizes using Cohen’s d for paired samples. We used inverse scores when analyzing responses for the distressing thought items that were framed positively (“Our team is united in taking care of this patient”) so that for all questions, higher scores would indicate higher distress. We did not correct for participants who completed multiple questionnaires corresponding to different interprofessional meetings.

## Results

### Participants

We collected 416 paired responses (80% participation rate) from 25 meetings spanning 2024–2025. This sample represented 192 unique clinicians (respondents attended an average of 2.1 meetings). An average of 21 clinicians attended each meeting. The largest faction (46%) had practiced 0-5 years. Nurses represented 76% of respondents, followed by physicians, advanced practice practitioners, child life/music therapists, and physical/occupational therapists (Table 1). The meetings were requested by clinicians from a range of professions and centered around diverse patient cases with a range of concerns (Supplementary Table 1).

**Table 1:** Characteristics of Participants.

Characteristic	(*N* = 416)
*Clinical Role*	**% of respondents**
Nurse	76%
Physician	6%
Advanced practice practitioner	6%
Social worker	5%
Child life/music therapist	3%
Physical/occupational therapist	3%
Chaplain	<1%
*Years in Practice*	
0–5	46%
6–10	19%
11–15	22%
16–20	5%
21–25	4%
26+	5%

### Conceptual model refinement

**Fig. 1:**
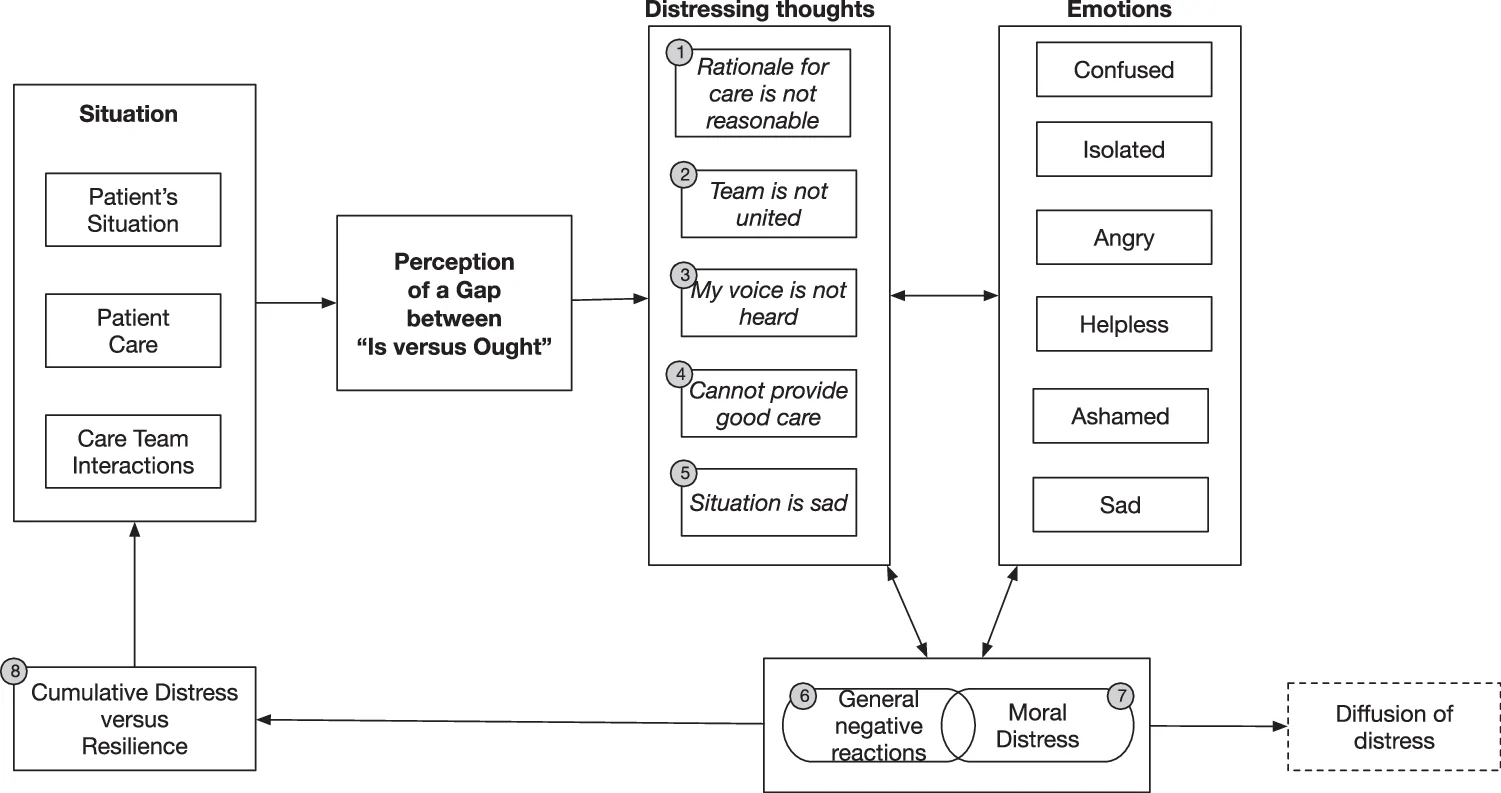
Conceptual model of distress. Our conceptual model both guided the development of and was iteratively revised through conducting our intervention. The model depicts distress as the product of a sequence of perceptions (specifically, a gap between what is and what ought to be), distressing thoughts (about how the current situation is not conforming to aspirational wishes), and feelings (arising from this lack of achievement of specific aspirations). These thoughts and feelings produce moral and general distress. Distress may be diffused through appropriate processing or result in residual negative reactions that impact perception in future cases. Numbers correspond to items in Table 2.

Qualitative information, gathered from meeting field notes, corroborated and expanded our conceptual model (Fig. 1). We revised the model to show that interactions with parents and between clinicians contributed to distress. Consistent with our initial model, distressing thoughts all in some way hinged on clinicians’ perception that something about the case was not as it ought to be. These thoughts, in turn, incited varied negative emotions and distress—moral and otherwise. Clinicians’ thoughts and reactions ranged from shame at feeling disrespected by a colleague to confusion about how parents were allowed to make an upsetting choice to sadness about the physical sight of a patient, which viscerally conveyed a grim prognosis. Clinicians also expressed simply wishing that situations were less sad. Throughout discussions, clinicians frequently referenced previous distressing cases and sources of resilience, which fed back to shape perceptions of the present case.tabfig

### Pre-meeting scores

During pilot testing, we made minimal wording changes, leaving questions largely unchanged from their validated derivations. Prior to meetings, clinicians agreed most strongly with the distressing thought “sad” (4.29, SD 0.72). The distressing thoughts garnering the next strongest agreement were: “rationale not reasonable” (1.93, SD 0.98), “not good care” (1.76, SD 0.97), “team not united” (1.56, SD 0.84), and “voice not heard” (1.51, SD 0.82). On pre-meeting questionnaires, mean moral distress was 5.5 (standard deviation [SD] 2.6) on the 10-point thermometer, or between uncomfortable and distressing, and general negative reactions were 5.5 (SD 2.3) (Table 2).

**Table 2:** Pre- and post-meeting scores.

Category	Pre-meeting mean (SD)	Post-meeting mean (SD)	*p*-value
*1- “The rationale for this patient’s plan is reasonable” (Likert)*	*1.93 (0.98)*	*1.52 (0.93)*	*<0.001*
*2- “Our team is united in taking care of this patient” (Likert)*	*1.56 (0.84)*	*1.22 (0.84)*	*<0.001*
*3- “I feel my voice is heard by the care team” (Likert)*	*1.51 (0.82)*	*1.17 (0.82)*	*<0.001*
*4- “I feel good about the care I am providing to this patient” (Likert)*	*1.76 (0.97)*	*1.62 (0.98)*	*<0.001*
5- “This patient’s situation makes me feel sad” (Likert)	4.31 (0.72)	4.30 (0.69)	0.39
*7- Moral Distress (thermometer)*	*5.47 (2.57)*	*5.27 (2.55)*	*0.005*
6- General Negative Reactions (thermometer)	5.52 (2.25)	5.44 (2.28)	0.1
8- “In general, my work is meaningful to me” (Likert)	4.29 (0.66)	–	–
“This meeting was worthwhile” (Likert)	–	4.16 (0.81)	–

Numbers correspond to the conceptual model. Italics indicate statistically significant change, *p* < 0.05.

### Score differences between disciplines

Prior to meetings, clinicians of all professions most strongly endorsed “sad,” but agreement with additional distressing thoughts varied substantially between professions. Nurses agreed most with “rationale not reasonable” (2.13, SD 0.95) and “not good care” (1.91, SD 0.93). Physicians agreed most with “team not united” (1.28, SD 0.94) and “rationale not reasonable” (1.16, SD 0.8). Social workers agreed most with “team not united” (1.42, SD 1.07) and “voice was not heard” (1.29, SD 0.90). General negative reactions were highest in nurses (mean 6.1, SD 1.9), followed by physicians (mean 4.0, SD 2.5), and advanced practice practitioners (mean 3.3, SD 2.0). Moral distress was also highest in nurses (mean 6.1, SD 2.1), followed by advanced practice practitioners (mean 3.8, SD 2.7) and physicians (mean 3.8, SD 2.5).

### Post-meeting scores

Most clinicians felt meetings were worthwhile (4.2, SD 0.83). Following meetings, clinicians reported reduced intensity of “rationale not reasonable” (1.52, SD 0.93, *p* < 0.001, Table 2), “not good care” (1.62, SD 0.98, *p* < 0.001), “team not united” (1.22, SD 0.84, *p* < 0.001), and “voice not heard” (1.17, SD 0.82, *p* < 0.001). Moral distress scores were lower after the meetings (5.27, SD 2.55, *p* = 0.005). There was no change in agreement with the case, making clinicians feel “sad” (*p* = 0.39), and meetings did not reduce general negative reactions (*p* = 0.10).

The effect size associated with meetings was medium for “rationale not reasonable” (Cohen’s d = 0.51), “team not united” (Cohen’s d = 0.38), and “voice not heard” (Cohen’s d = 0.39) and small for “not good care” (Cohen’s d = 0.19) and moral distress (Cohen’s d = 0.14). Across these questions, meetings had a greater impact on respondents who reported high distress prior to meetings. For the subgroup of participants who agreed or strongly agreed with “rationale not reasonable,” “team not united,” and “voice not heard” in their pre-meeting questionnaires, the impact on these questions had a very large effect size (Cohen’s d = 1.10, 1.17, 1.16, respectively). Finding work meaningful did not moderate the impact of meetings.

## Discussion

In response to challenging cases, clinicians reported common distressing thoughts, which varied by profession. Interprofessional meetings convened on an as-needed basis appear to have reduced the intensity of distressing thoughts about the cases, and clinicians found these meetings worthwhile. After meetings, clinicians were more likely to report that their perspective was heard, the team was united, they could provide good care, and the rationale for care was reasonable. They also reported lower moral distress. Pre-to-post meeting change was greatest for clinicians with high levels of distress related to the case discussed.

Our program for interprofessional meetings worked primarily by creating “moral space,” [33] a forum for reflection and conversation, rather than clarifying right from wrong. In the modern ICU, bustling with constant tasks and rotating clinicians [9], perhaps the most important function of meetings was providing a shared pause. The change in pace from minute-to-minute tasks to slowed reexamination was key to any benefits conferred by the program. The existence of the program itself and the ability of any individual clinician to request a meeting signaled that all team members’ perspectives were valued. Meetings facilitated communication across disciplines and provided solidarity in revealing that many thoughts and feelings were shared broadly. This open-endedness distinguishes our program from many preceding interventions [14, 34, 35]. Our meetings provided as-needed real-time support targeting specific cases, irrespective of distress type, rather than formulaic longitudinal learning directed toward a specific type of distress.

Both formulaic and open-ended programs have advantages and drawbacks, and we see three principal advantages of our open-ended approach. First, the open-ended approach enabled clinicians to request meetings on an as-needed basis directed support, in real time, to the cases clinicians found most troubling. This selection process resembles “hot” debriefs immediately following acute clinical events, which have been shown to decrease both short-term distress and longer-term sequelae [36–38]. That our data suggest meetings were most effective in mitigating distress when levels were high further supports the benefits of real-time support. Second, our meetings accommodated discussion of a range of distressing thoughts and responses, customizing the focus to the individual circumstances and often including discussion of several distressing thoughts in a single meeting. This flexibility allowed clinicians to discuss multiple distressing thoughts, which impact clinicians from different disciplines variably. Clarifying the type of distress people were experiencing was likely one way that conversations were beneficial. Additionally, discussing an active case may enhance retention of any lessons clinicians take away, as making education salient to current lived experience is a tenet of adult education [39]. Third, flexibility in the type of distress discussed also enabled flexibility in response. For example, through conversations, clinicians sometimes realized that their concerns were at least partially rooted in practical difficulties. Practical challenges may be more easily addressed than moral or existential ones [14]. Of course, given the multifaceted nature of distress, many strategies with many different strengths will be required to sufficiently support clinicians.

Our approach also has three main drawbacks. First, we did not evaluate the long-term impact of meetings. While meetings reduced the intensity of distressing thoughts in the short-term, itself a benefit, we cannot assess their longer-term impact on clinicians’ experiences with the cases discussed or other similar future cases. Our program did not impart specific training, unlike curricula focused on particular types of distress that teach ethical frameworks, for example [17, 40, 41]. We are hopeful that challenging and reducing distressing thoughts collectively may provide clinicians with lasting mechanisms for such alleviation, again akin to the long-term efficacy of cognitive behavioral therapy [24, 30], but this remains unproven. Future research may also explore whether such meetings have a downstream impact on patient care. Along the same lines, the significance of our finding that meetings did not decrease sadness or general negative reactions will require further study. We hypothesize that troubling cases, which frequently end in death or other tragedy, remain sad even when other elements of team dynamics or moral distress may be reduced, yet we cannot say how this continued sadness impacts clinicians. A second limitation is that attendance at meetings was voluntary (except for the attending physician and social worker), so we cannot rule out the possibility that those more likely to benefit may have self-selected to participate. We cannot accurately calculate an attendance rate because we used email listservs to distribute the meeting invitations and scheduled meetings during daytime work hours, limiting the ability of night-shift clinicians to attend. Further work will be needed to determine how to support clinicians who do not attend these meetings, as well as any subgroup that experiences less benefit from meetings. Third, and finally, future work will be needed to evaluate the generalizability of our program to other centers with different facilitators.

Despite limitations, our program seems to have been helpful to clinicians. The growth of our program over time and the large number of clinicians who voluntarily attend meetings attest to the perceived usefulness. Further, that most clinicians reported meetings to be worthwhile—worth using 45 min of busy clinical days to attend—supports the benefit. Compared to many studies on clinician distress, our sample size is large, and our design is rigorous [14, 34, 35]. A program of responsive, as-needed interprofessional meetings represents a feasible, effective strategy that has promise to help clinicians in varied settings [42–44].

## Conclusion

Interprofessional meetings held on a timely, as-needed basis appear to have decreased numerous distressing thoughts and moral distress among clinicians from many professions. Meetings helped clinicians feel their perspective was heard, the team was united, they could do a good job, and the rationale for care was reasonable.

## Supplementary information

Supplementary Material

## Data Availability

Deidentified data will be shared via email to the corresponding author.

## References

[1] Austin CL, Saylor R, Finley PJ. Moral distress in physicians and nurses: Impact on professional quality of life and turnover. Psychol Trauma. 2017;9:399–406. 10.1037/tra0000201. 27797570

[2] Prentice TM, Janvier A, Gillam L, Donath S, Davis PG. Moral distress in neonatology. Pediatrics. 2021;148:e2020031864 10.1542/peds.2020-031864. 34285081

[3] Jameton A. Nursing practice: the ethical issues. Englewood Cliffs, N.J.: Prentice-Hall; 1984.

[4] Morley G, Ives J, Bradbury-Jones C, Irvine F. What is “moral distress”? A narrative synthesis of the literature. Nurs Ethics. 2019;26:646–62. 10.1177/0969733017724354. PMC6506903 28990446

[5] Costa DK, Moss M. The cost of caring: emotion, burnout, and psychological distress in critical care clinicians. Ann Am Thorac Soc. 2018;15:787–90. 10.1513/AnnalsATS.201804-269PS. PMC6207112 29727197

[6] Dean W, Talbot SG, Caplan A. Clarifying the language of clinician distress. JAMA. 2020;323:923–4. 10.1001/jama.2019.21576. 32003769

[7] Callahan KP, Taha D, Dewitt A, Munson DA, Behringer K, Feudtner C. Clinician distress with treatments at the frontier of mortality.J Pediatr. 2022. 10.1016/j.jpeds.2022.09.013 PMC10251120 36115624

[8] Kesewa Layman AA, Press Callahan K, Nathanson P, Lechtenberg L, Hill D, Feudtner C. Simple interventions for pediatric residents’ moral distress: a randomized, controlled experiment. Pediatrics. 2023;151:e2022060269 10.1542/peds.2022-060269. PMC10416266 37153965

[9] Leland BD, Wocial LD, Madrigal VN, et al. Group concept mapping conceptualizes high-quality care for long-stay pediatric intensive care unit patients and families. J Pediatr. 2023;252:48–55.e1. 10.1016/j.jpeds.2022.08.007. PMC9772094 35973447

[10] Sorensen R, Iedema R. Emotional labour: clinicians’ attitudes to death and dying. J Health Organ Manag. 2009;23:5–22. 10.1108/14777260910942524. 19455875

[11] Henrich NJ, Dodek PM, Gladstone E, et al. Consequences of moral distress in the intensive care unit: a qualitative study. Am J Crit Care. 2017;26:e48–e57. 10.4037/ajcc2017786. 28668926

[12] Vahey DC, Aiken LH, Sloane DM, Clarke SP, Vargas D. Nurse burnout and patient satisfaction. Med Care. 2004;42:II57–66. 10.1097/01.mlr.0000109126.50398.5a. PMC2904602 14734943

[13] Baer TE, Feraco AM, Tuysuzoglu Sagalowsky S, Williams D, Litman HJ, Vinci RJ. Pediatric resident burnout and attitudes toward patients. Pediatrics. 2017;139:e20162163 10.1542/peds.2016-2163. 28232639

[14] Prentice TM, Gillam L, Davis PG, Janvier A. The use and misuse of moral distress in neonatology. Semin Fetal Neonatal Med. 2018;23:39–43. 10.1016/j.siny.2017.09.007. 28964686

[15] Delany C, Jones S, Sokol J, Gillam L, Prentice T. Reflecting before, during, and after the heat of the moment: a review of four approaches for supporting health staff to manage stressful events. Bioethical Inq. 2021;18:573–87. 10.1007/s11673-021-10140-0. 34741699

[16] Abbasi S, Ghafari S, Shahriari M, Shahgholian N. Effect of moral empowerment program on moral distress in intensive care unit nurses. Nurs Ethics. 2019;26:1494–504. 10.1177/0969733018766576. 29695198

[17] Beumer CM. Innovative solutions: the effect of a workshop on reducing the experience of moral distress in an intensive care unit setting. Dimens Crit Care Nurs. 2008;27:263–7. 10.1097/01.DCC.0000338871.77658.03. 18953194

[18] Leggett JM, Wasson K, Sinacore JM, Gamelli RL. A pilot study examining moral distress in nurses working in one United States burn center. J Burn Care Res. 2013;34:521–8. 10.1097/BCR.0b013e31828c7397. 23884046

[19] Rogers S, Babgi A, Gomez C. Educational interventions in end-of-life care: part I: an educational intervention responding to the moral distress of NICU nurses provided by an ethics consultation team. Adv Neonatal Care. 2008;8:56–65. 10.1097/01.ANC.0000311017.02005.20. 18300739

[20] Browning ED, Cruz JS. Reflective debriefing: a social work intervention addressing moral distress among ICU nurses. J Soc Work End Life Palliat Care. 2018;14:44–72. 10.1080/15524256.2018.1437588. 29488856

[21] Dawson J, McCarthy I, Taylor C, et al. Effectiveness of a group intervention to reduce the psychological distress of healthcare staff: a pre-post quasi-experimental evaluation. BMC Health Serv Res. 2021;21:392. 10.1186/s12913-021-06413-4. PMC8076663 33906664

[22] Imbulana DI, Davis PG, Prentice TM. Interventions to reduce moral distress in clinicians working in intensive care: a systematic review. Intensive Crit Care Nurs. 2021;66:103092 10.1016/j.iccn.2021.103092. 34147334

[23] Mitchell MD, Gehrman P, Perlis M, Umscheid CA. Comparative effectiveness of cognitive behavioral therapy for insomnia: a systematic review. BMC Fam Pract. 2012;13:40 10.1186/1471-2296-13-40. PMC3481424 22631616

[24] Hollon SD, Beck AT. Cognitive and cognitive-behavioral therapies. In: Handbook of psychotherapy and behavior change, 4th Ed. John Wiley & Sons; Englewood Cliffs, N.J.: Prentice-Hall; 1994.

[25] Gibbs, G. Learning by doing: A guide to teaching and learning methods. Further Educational Unit, Oxford Polytechnic, Oxford. - References - Scientific Research Publishing. 2025. https://www.scirp.org/reference/referencespapers?referenceid=478921

[26] Reflective practice groups and nurse professional quality of life. Aus J Adv Nurs. 2025. https://search.informit.org/doi/abs/10.3316/informit.191522643999356

[27] Wocial LD, Weaver MT. Development and psychometric testing of a new tool for detecting moral distress: the Moral Distress Thermometer. J Adv Nurs. 2013;69:167–74. 10.1111/j.1365-2648.2012.06036.x. 22607094

[28] Harris PA, Taylor R, Minor BL, et al. The REDCap consortium: building an international community of software platform partners. J Biomed Inf. 2019;95:103208 10.1016/j.jbi.2019.103208. PMC7254481 31078660

[29] McKenna L, Handscomb L, Hoare DJ, Hall DA. A scientific cognitive-behavioral model of tinnitus: novel conceptualizations of tinnitus distress. Front Neurol. 2014;5:196 10.3389/fneur.2014.00196. PMC4186305 25339938

[30] Nakao M, Shirotsuki K, Sugaya N. Cognitive–behavioral therapy for management of mental health and stress-related disorders: recent advances in techniques and technologies. Biopsychosoc Med. 2021;15:16. 10.1186/s13030-021-00219-w. PMC8489050 34602086

[31] Batuchina A, Iždonaitė-Medžiūnienė I, Lecaj R. Multidimensional scale of meaningful work: construction and validation. Front Psychol. 2025;16:1578825 10.3389/fpsyg.2025.1578825. PMC11961940 40177034

[32] Yurek LA, Vasey J, Sullivan Havens D. The use of self-generated identification codes in longitudinal research. Eval Rev. 2008;32:435–52. 10.1177/0193841X08316676. 18477737

[33] Walker MU. Keeping moral space open. New images of ethics consulting. Hastings Cent Rep. 1993;23:33–40. 8463088

[34] Morley G, Field R, Horsburgh CC, Burchill C. Interventions to mitigate moral distress: a systematic review of the literature. Int J Nurs Stud. 2021;121:103984 10.1016/j.ijnurstu.2021.103984. 34214894

[35] Kalani SD, Azadfallah P, Oreyzi H, Adibi P. Interventions for physician burnout: a systematic review of systematic reviews. Int J Prev Med. 2018;9:81 10.4103/ijpvm.IJPVM_255_18. PMC6151971 30283613

[36] Gilmartin S, Martin L, Kenny S, Callanan I, Salter N. Promoting hot debriefing in an emergency department. BMJ Open Qual. 2020;9. 10.1136/bmjoq-2020-000913 PMC7430325 32816864

[37] Alanezi FZ, Miller E, Morrison CF, Kelcey B, Wagner R. Hot versus cold debriefing in a nursing context: an integrative review. J Nurs Educ. 2024;63:653–8. 10.3928/01484834-20240529-02. 39388470

[38] Knobler HY, Nachshoni T, Jaffe E, Peretz G, Yehuda YB. Psychological guidelines for a medical team debriefing after a stressful event. Mil Med. 2007;172:581–5. 10.7205/milmed.172.6.581. 17615836

[39] Adult Learning Principles. 2025. https://www.cdc.gov/training-development/media/pdfs/2024/04/adult-learning-principles.pdf

[40] Allen R, Butler E. Addressing moral distress in critical care nurses: a pilot study. Int J Crit Care Emerg Med. 2016;2. https://scholarlycommons.baptisthealth.net/se-all-publications/1151

[41] Monteverde S. Caring for tomorrow’s workforce: Moral resilience and healthcare ethics education. Nurs Ethics. 2016;23:104–16. 10.1177/0969733014557140. 25505083

[42] National Academies of Sciences, Engineering, and Medicine; National Academy of Medicine; Committee on Systems Approaches to Improve Patient Care by Supporting Clinician Well-Being. Taking Action Against Clinician Burnout: A Systems Approach to Professional Well-Being. National Academies Press (US); 2019. 2025. http://www.ncbi.nlm.nih.gov/books/NBK552618/

[43] Awa WL, Plaumann M, Walter U. Burnout prevention: a review of intervention programs. Patient Educ Counseling. 2010;78:184–90. 10.1016/j.pec.2009.04.008. 19467822

[44] van Mol MMC, Kompanje EJO, Benoit DD, Bakker J, Nijkamp MD. The prevalence of compassion fatigue and burnout among healthcare professionals in intensive care units: a systematic review. PLoS One. 2015;10:e0136955 10.1371/journal.pone.0136955. PMC4554995 26322644

