# Supplementary material for: Addressing neonatal intensive care unit clinicians’ distress via interprofessional meetings
Source: J Perinatol. 2026 Jun 22;46(11):2166–71. doi: 10.1038/s41372-026-02763-1 (PMC13731130; doi:10.1038/s41372-026-02763-1)
Supplement: Supplementary file 1 [file 41372_2026_2763_MOESM1_ESM.docx]

**Supplemental Figure 1. Pink Flags Meeting Guide**

1. Introduction of moderator and review of agenda
2. Pause (30 seconds) to center ourselves and honor the effort we all put into our work
3. Perspective of medical team—presented by attending physician
4. Perspective of social work team—presented by following social worker
5. Summary of known concerns/challenges—presented by moderator
6. Climate at bedside—opportunity for nursing team to comment
7. Moderated discussion—open to everyone to comment and respond
   a. Participants encouraged to share why the case is challenging
   b. Participants also encouraged to contribute ideas about what might help improve the situation or alleviate distress
8. Summary by moderator—review reasons for distress and any potential solutions discussed

**Supplemental Figure 2. Pre-Meeting Questionnaire**

You are being asked to take part in this research study because you are a clinician attending a Pink Flags meeting. We are hoping to learn about whether these meetings are helpful in reducing distress. There are minimal risks from this study, which include a possible breach of confidentiality. However, your responses are de-identified and cannot be linked back to you. Participation is voluntary and will take 3-5 minutes in total. You will be compensated for your time with a $10 Amazon gift card but will not benefit directly from the study. Completion of this study implies consent. If you are a CHOP employee, your decision to participate will not be shared with your supervisor and will not have any effect on your performance evaluation or employment status. Please do not hesitate to contact us with questions by email (callahankp@ chop.edu) or phone (215-275-6094).

***Note: References were omitted from the actual questionnaire***

**Please rate your overall distress (any negative reactions) about this patient case**^1,2^

**Please rate your moral distress (feeling you know the right thing to do but are unable to do it) about this patient case**^1,2^

**To what extent do you agree with the following statements?**

**This patient’s situation makes me feel sad.**^3–7^

Strongly disagree--- Disagree--- Neutral--- Agree--- Strongly Agree

**I feel my voice is heard by the care team.**^8–10^

Strongly disagree--- Disagree--- Neutral--- Agree--- Strongly Agree

**I feel good about the care I am providing to this patient.**^3,11,12^

Strongly disagree--- Disagree--- Neutral--- Agree--- Strongly Agree

**The rationale for this patient’s care plan is reasonable.** ^9^

Strongly disagree--- Disagree--- Neutral--- Agree--- Strongly Agree

**Our team is united in taking care of this patient.**^9^

Strongly disagree--- Disagree--- Neutral--- Agree--- Strongly Agree

**In general, my work is meaningful to me.**^8,13^

Strongly disagree--- Disagree--- Neutral--- Agree--- Strongly Agree

**The next few questions let us anonymously link your pre- and post- meeting surveys.**

- Second letter of your first name:
- Number representing the month you were born:
- First letter of mother’s first name:
- Number of older siblings:

**Supplemental Figure 3. Post-Meeting Questionnaire**

Thank you for coming to a Pink Flags meeting. We so appreciate your input as we continue to develop this program.

***Note: References were omitted from the actual questionnaire***

**Please rate your overall distress (any negative reactions) about this patient case**^1,2^

**Please rate your moral distress (feeling you know the right thing to do but are unable to do it) about this patient case**^1,2^

**To what extent do you agree with the following statements?**

**This patient’s situation makes me feel sad.**^3–7^

Strongly disagree--- Disagree--- Neutral--- Agree--- Strongly Agree

**I feel my voice is heard by the care team.**^8–10^

Strongly disagree--- Disagree--- Neutral--- Agree--- Strongly Agree

**I feel good about the care I am providing to this patient.**^3,11,12^

Strongly disagree--- Disagree--- Neutral--- Agree--- Strongly Agree

**The rationale for this patient’s care plan is reasonable.** ^9^

Strongly disagree--- Disagree--- Neutral--- Agree--- Strongly Agree

**Our team is united in taking care of this patient.**^9^

Strongly disagree--- Disagree--- Neutral--- Agree--- Strongly Agree

**This meeting was worthwhile.**

Strongly disagree--- Disagree--- Neutral--- Agree--- Strongly Agree

**The next few questions let us anonymously link your pre- and post- meeting surveys.**

- Second letter of your first name:
- Number representing the month you were born:
- First letter of mother’s first name:
- Number of older siblings:

**Specify clinical role:**

**Years in practice:**

**Supplemental Table 1. Patient diagnosis, profession of requestor, and reason for request for each meeting.**

| **Meeting ID** | **Patient Diagnosis** | **Profession of Meeting Requestor** | **Reason for Request** |
| --- | --- | --- | --- |
| 1 | Metabolic disease | Clinical nurse | Concern about communication with family |
| 2 | Chronic lung disease | Clinical supervisor (RN) | Concerns about family understanding; concerns about family's treatment of bedside team |
| 3 | Holoprosencephaly | Social worker | Concern patient is suffering, concerns about 'futility' |
| 4 | Chronic lung disease | Clinical nurse | Parents impeding RN work flow, concern patient not comfortable, concerns about intra-team communication |
| 5 | Chronic lung disease, lymphatic dysfunction | Clinical nurse | Concern for discomfort of patient; challenges partnering with parent with mental illness |
| 6 | Extremely preterm | Clinical nurse | Practical difficulties in care, concerns about communication with parents |
| 7 | Skeletal dysplasia | Clinical nurse educator | Request for clarification of medical plan; concerns about communication within team and with family |
| 8 | Congenital urogenital anomaly | Clinical supervisor (RN) | Team requesting clarity about goals of care; ongoing sad situation |
| 9 | Numerous sequelae of prematurity | Clinical nurse | Concern baby is suffering |
| 10 | Chronic lung disease | Clinical supervisor (RN) | Concern that family is struggling to cope; ongoing sadness among clinicians |
| 11 | Chronic lung disease | Clinical supervisor (RN) | Concern about communication among team; request for clarification about parent's visitation restriction |
| 12 | Congenital diaphragmatic hernia | Front line/Nurse practitioner | Concerns about appropriateness of medical treatment plan; concerns about communication |
| 13 | Hypoxic ischemic encephalopathy | Clinical nurse | Concerns about 'futility'; requests for practical support for clinicians |
| 14 | Multiple congenital anomalies | Clinical nurse | Concerns about family coping |
| 15 | Skeletal dysplasia | Occupational therapist | practical care challenges; concerns about communication with family |
| 16 | Holoprosencephaly | Clinical nurse | Concern baby is suffering; concerns about communication among team |
| 17 | Skeletal dysplasia | Clinical nurse educator | Request for clarification of medical plan; concerns about communication within team and with family |
| 18 | Trisomy 18 | Nurse manager | Concerns about social situation of family; concerns about 'futility' |
| 19 | Congenital surfactant deficiency | Clinical nurse | Concerns about intra-team communication |
| 20 | Skeletal dysplasia | Clinical nurse | Request for clarification of medical plan; concerns about family understanding |
| 21 | Hypoxic ischemic encephalopathy | Clinical nurse educator | Concern baby is suffering; concerns about 'futility' |
| 22 | Hydrocephalus | Clinical nurse | Request for clarification about goals of care; ongoing sad situation |
| 23 | Holoprosencephaly | Nurse manager | Concerns about 'futility; |
| 24 | Metabolic disease | Clinical nurse | Concerns about communication with family; challenging clinical care |
| 25 | Skeletal dysplasia | Clinical nurse | Challenging care coordination; concerns that care not helping baby |

**References**

1.	Wocial LD, Weaver MT. Development and psychometric testing of a new tool for detecting moral distress: the Moral Distress Thermometer. *J Adv Nurs*. 2013;69(1):167-174. doi:10.1111/j.1365-2648.2012.06036.x

2.	Wocial L, Ackerman V, Leland B, et al. Pediatric Ethics and Communication Excellence (PEACE) Rounds: Decreasing Moral Distress and Patient Length of Stay in the PICU. *HEC Forum*. 2017;29(1):75-91. doi:10.1007/s10730-016-9313-0

3.	Henry SG, Fenton JJ, Campbell CI, et al. Development and Testing of a Communication Intervention to Improve Chronic Pain Management in Primary Care: A Pilot Randomized Clinical Trial. *Clin J Pain*. 2022;38(10):620-631. doi:10.1097/AJP.0000000000001064

4.	Ammentorp J, Sabroe S, Kofoed PE, Mainz J. The effect of training in communication skills on medical doctors’ and nurses’ self-efficacy. A randomized controlled trial. *Patient Educ Couns*. 2007;66(3):270-277. doi:10.1016/j.pec.2006.12.012

5.	Verduyn P, Tuerlinckx F, Gorp K. Measuring the duration of emotional experience: The influence of actual duration and response format. *Quality & Quantity*. 2012;47. doi:10.1007/s11135-012-9671-x

6.	Alshutwi M, Alawad M, Alammari M, et al. Perceived impact of patients’ suicide and serious suicidal attempts on their treating psychiatrists and trainees: a national cross-sectional study in Saudi Arabia. *BMC Psychiatry*. 2023;23(1):607. doi:10.1186/s12888-023-05042-x

7.	Frintner MP, Leslie LK, Gottschlich EA, Starmer AJ, Byrne BJ, Freed GL. Pediatricians’ Career Satisfaction and Wellbeing by Sex Before and During the COVID-19 Pandemic. *Pediatrics*. 2024;153(3):e2023063345. doi:10.1542/peds.2023-063345

8.	Smith S, Goldhaber N, Maysent K, Lang U, Daniel M, Longhurst C. Impact of a virtual coaching program for women physicians on burnout, fulfillment, and self-valuation. *BMC Psychol*. 2024;12(1):331. doi:10.1186/s40359-024-01763-0

9.	Trockel M, Bohman B, Lesure E, et al. A Brief Instrument to Assess Both Burnout and Professional Fulfillment in Physicians: Reliability and Validity, Including Correlation with Self-Reported Medical Errors, in a Sample of Resident and Practicing Physicians. *Acad Psychiatry*. 2018;42(1):11-24. doi:10.1007/s40596-017-0849-3

10.	Kiser SB, Sterns JD, Lai PY, Horick NK, Palamara K. Physician Coaching by Professionally Trained Peers for Burnout and Well-Being: A Randomized Clinical Trial. *JAMA Netw Open*. 2024;7(4):e245645. doi:10.1001/jamanetworkopen.2024.5645

11.	Dunlay SM, Foxen JL, Cole T, et al. A survey of clinician attitudes and self-reported practices regarding end-of-life care in heart failure. *Palliat Med*. 2015;29(3):260-267. doi:10.1177/0269216314556565

12.	Angel E, Craven R, Denson N. The Nurses Self-Concept Instrument (NSCI): assessment of psychometric properties for Australian domestic and international student nurses. *Int J Nurs Stud*. 2012;49(7):880-886. doi:10.1016/j.ijnurstu.2012.01.016

13.	Batuchina A, Iždonaitė-Medžiūnienė I, Lecaj R. Multidimensional scale of meaningful work: construction and validation. *Front Psychol*. 2025;16:1578825. doi:10.3389/fpsyg.2025.1578825
